# Crowding reduces per-capita parasite infection risk in a butterfly host

**DOI:** 10.1098/rspb.2025.1110

**Published:** 2025-09-10

**Authors:** Ania A. Majewska, Richard J. Hall, Jacobus C. de Roode

**Affiliations:** ^1^ Southeastern Cooperative Wildlife Disease Study, Population Health, College of Veterinary Medicine, University of Georgia, Athens, GA, USA; ^2^ Physiology and Pharmacology, College of Veterinary Medicine, University of Georgia, Athens, GA, USA; ^3^ Center for the Ecology of Infectious Disease, University of Georgia, Athens, GA, USA; ^4^ Odum School of Ecology, University of Georgia, Athens, GA, USA; ^5^ Infectious Diseases, College of Veterinary Medicine, University of Georgia, Athens, GA, USA; ^6^ Department of Biology, Emory University, Atlanta, GA, USA

**Keywords:** host density, dilution effect, encounter reduction, parasite burden, environmental transmission, monarch

## Abstract

Crowding can result in greater disease transmission, yet crowded hosts may also remove infectious propagules from the environment, thereby lowering the encounter rate and infectious dose received by conspecifics. We combined experimental and modelling work to examine the impact of crowding of butterfly larvae on the per-capita risk of infection by a protozoan that is transmitted via the larval food plant, and the resulting infection load in adult butterflies. We reared larvae at different densities and exposed them to low and high doses of parasites. We modified an existing model to include effects of conspecific density on food (and thus parasite) consumption rate and infected adult mortality rate. Experimental work indicated that the proportion of infected hosts on plants with ten caterpillars were reduced by at least 50% compared with single caterpillars. High density reduced per-capita infection risk and parasite load and extended lifespan of all hosts, as crowded hosts removed parasites from the environment. Modelling suggested that the lower consumption rate due to crowding can lower infection prevalence by as much as 20%, although the number of new cases increases with larger population size. Our results highlight that the expected positive relationship between host density and infection prevalence breaks down when crowding results in removal of infectious propagules from the environment.

## 1. Introduction

Host density is one of the most important drivers of infectious disease transmission in populations. For parasites transmitted through close proximity to infected hosts, high host densities are expected to increase transmission rates due to greater contact rates between infectious and susceptible individuals [1,2]. Crowded populations can therefore experience higher prevalence of infected individuals, as has been shown across mammals, birds and insects [3–7]. A positive relationship between host density and infection prevalence is not restricted to directly transmitted diseases (i.e. spread via direct contact). For indirectly transmitted diseases, such as those spread via a vector or free-living propagules in the environment, crowding can result in greater infection rates of vectors or deposition of infectious propagules in the environment, thereby increasing between-host transmission [8–10]. For example, high density of white-tailed deer in urbanized areas is associated with an increased prevalence of chronic wasting disease, probably due to prion-contaminated soil which deer consume during grazing, grooming and mineral licking [11,12].

For pathogens with environmental transmission, the expectation that higher host densities result in greater parasite transmission is based on the assumption that contact with the parasite in the environment scales with density via mass action [13–15]. High host densities can result in intensified deposition of free-living infectious stages into the environment [1,16,17], which then results in a high likelihood that susceptible hosts encounter them—or that they encounter high doses and are thus more likely to be infected. However, several mechanisms can lead to the opposite effect, where higher host densities result in lower per capita risk of infection [18–20]. Hosts at high densities can interfere with other hosts’ foraging and consumption via territorial, physical and chemical interactions, resulting in the *host foraging interference* effect [21–23]. At the same time, with high host density, infective stages in the environment are divided among many hosts, resulting in *encounter dilution* effect [24] (also referred to as *safety in numbers*) and lower population-level infection prevalence. Encounter dilution can also occur when hosts compete for the same food resources and competitors remove infectious free-living propagules from the environment (e.g. via consumption) and thereby lower per capita infection risk for other hosts [25–28]. The encounter effect is particularly notable for parasites with complex life cycles, such as trematodes, where the number of infective stages deposited in a patch is limited and transmission is decoupled in space or time [29–31]. Whether it is interference of foraging or decrease in the number of parasites in the environment, both mechanisms result in reduced incidence of consuming the parasite and consequent infection. Indeed, theoretical work by Civitello *et al.* [23] showed that both host foraging interference and encounter dilution can reduce epidemics.

High host density can alter the per capita risk of infection by changing the density of parasites in the environment, which consequently alters the dose of parasites that each host acquires [32]. Indeed, in various host–parasite systems with free-living parasite stages, including reindeer and warble flies, and sea lice and salmon, hosts experience lower individual risk of being 'attacked' by a parasite and lower parasite burden as host density increases [33,34]. Similar trends are expected for non-mobile free-living parasites that depend on host movement and foraging for encounters: high host density decreases the total number of parasites in the environment via consumption, resulting in fewer hosts with high-intensity infections and more hosts with intermediate and low intensities. In such a case, when high host density is linked to encounter dilution, it can lower the negative impacts of infection on individual and population levels.

In this study, we combined experimental work and mathematical modelling to examine the role of high host density and associated host foraging interference and encounter dilution, on per capita infection risk within a single species that consumes the infectious stages from the environment. For the study system examined here, we are unable to distinguish whether changes in infection risk are due to host foraging interference or encounter dilution, and therefore refer to the two effects collectively as *foraging dilution*. We focused the work on the interaction between the monarch butterfly (*Danaus plexippus*) and its protozoan parasite *Ophryocystis elektroscirrha* (OE). Infection with OE can be debilitating or lethal for monarchs; the parasite causes reductions in mating success, fecundity, flight ability and lifespan, with worsening fitness consequences experienced with greater doses of ingested OE spores [35–37]. One study estimated reduction of the adult population size by 50% when infection rates are high [38]. Population level infection rates are therefore of concern and the increasing levels of infection in the eastern monarch may be contributing to the loss of monarchs during autumn migration [39].

This system is ideal for examining questions about density and per-capita infection risk because monarchs scatter infectious parasite stages onto milkweed plants, the food resource for their caterpillars. An OE infection starts when a caterpillar ingests spores while consuming milkweed, and a single parasite spore can cause an infection [40]. In the field, three transmission routes have been identified: vertical transmission (larvae infected by spores shed onto their egg cases by their infected mother) [41], environmental transmission (larvae consume spore-contaminated milkweed leaves) and adult transfer (larvae infected by spores shed onto their egg cases by adults contaminated with spores through mating with infected adults) [38]. Once consumed, parasites penetrate the mid-gut, replicate internally, and adults emerge covered in millions of dormant spores [40,42]. The monarch does not have gregarious larval stages (i.e. eggs are laid singly), with average densities of around 0.04 eggs per plant across the main breeding range at the peak months [43], although densities are higher at sites where non-native milkweed, *Asclepias curassavica* is planted [44].

For this work, we first carried out two experiments in which we manipulated the density of monarch caterpillars on milkweed and parasite load on experimental plants as occurs in breeding sites with *Asclepias curassavica* over the breeding season [38]. We measured host infection rates, infection loads, adult lifespan and morphology. We expected that exposure to higher parasite doses (within equivalent host density treatments) would result in a greater number and proportion of infected monarchs; and that high caterpillar density (with equivalent dose treatments) would lower infection probability and resulting parasite load, as would occur with foraging dilution. As predicted, we found that increased parasite dose resulted in greater per-capita infection risk; moreover, crowding of caterpillars on plants (rearing at high density) benefited individuals as it lowered individual infection risk, reduced parasite spore load and increased lifespan.

Next, to examine how foraging dilution impacts population level infection dynamics, we modified a mechanistic mathematical model of the monarch–OE system [38]. To simulate foraging dilution (lower chances of consuming a leaf with an OE spore and therefore risk of infection), we varied the per capita leaf consumption rates (an integral part of the previously developed model) and explored how this influenced population-level infection prevalence. Given the expected impact of larvae consuming less milkweed, we assessed how the relative contribution of the three transmission routes changed over time. Finally, because reduced spore consumption leads to adults infected with lower parasite loads and consequently lower disease-induced mortality, we also examined infection dynamics given longer lifespan of infected adults. The model was parameterized for *Asclepias curassavica* milkweed sites, where densities of caterpillars and infection rates are high [38].

## 2. Material and methods

### (a) Experimental study

We used six distinct non-inbred genetic monarch families, the grand-progeny of eastern North American monarchs collected at St Marks, Florida, USA in October 2019 and overwintered in the laboratory. Mating and egg laying occurred in controlled environmental chambers with lights set to a 16 h photoperiod and temperature set to 26°C, during June and July 2020 in Atlanta, Georgia, USA. Eggs were collected on greenhouse-reared milkweed plants *Asclepias curassavica*. We transferred 2nd instar stage larvae to milkweed plants covered with a clear plastic tube (10 cm diameter, 61 cm height) and mesh cap. We randomly assigned larvae from the different families to each of the density treatments: in experiment 1 singles (one caterpillar per plant) and tens (ten caterpillars per plant); in experiment 2: singles (one caterpillar per plant), doubles (two caterpillars per plant), and tens (ten caterpillars per plant). The two experiments differed in how spores were deposited onto the plant, with lower exposure in the first experiment than in the second experiment, to capture the range of parasites monarchs are expected to be exposed to in nature. Specifically, in experiment 1, spores (parasite source: P44) were deposited on the top leaves of milkweed plants prior to placement of larvae on the plants and dose exposure consisted of 0 spores (control), 10 spores and 100 spores. In experiment 2, we used a single perished highly infected adult to deposit spores on the top leaves of milkweed plants by touching the leaves once for low dose treatment, and ten times for high dose treatment. Previous work suggests that highly infected adults are covered with millions of infectious spores and can deposit 100 to 1000 spores per leaf at each contact [36]. Therefore, we assume that a low dose in experiment 2 consisted of exposure to 100−1000 spores, while the high dose consisted of exposure to 1000–10 000 spores. In experiment 1 we included 25 replicates of singles and 6 replicates of tens of each exposure dosage (control, in low dose, high dose). In experiment 2, we included 20 replicates of singles, 15 of doubles and 8 of tens of each exposure. A total of 499 caterpillars were used at the initiation of the study (experiment 1: *n* = 259, experiment 2: *n* = 240). For sample sizes, see electronic supplementary material, tables S1 and S7.

We provided the larvae with new plants, not inoculated with spores, once leaves on the top two thirds of the inoculated plant were consumed. This ensured that the deposited spores on the inoculated plant were consumed and the larvae did not experience food limitation, which can negatively impact adult size and caterpillar survival [45]. Given that in the wild, monarch caterpillars abandon the original plant once it is substantially reduced in foliage in search of new plants [46], we assume replacing plants mimicked natural settings. Further, we replaced plants because the goal of the experiments was to investigate the role of density, not food limitation, on parasite infection and burden. Upon pupation, individuals were transferred to 16 oz (473 ml) plastic SOLO cups. Adults were held in glassine envelopes in an incubator at 12°C and checked daily to measure lifespan. To estimate parasite load, upon monarch death, we vortexed the abdomens in 5 ml of tap water for 5 min and quantified the number of spores with a haemocytometer slide following [37]. To meet model assumptions of normality of error distributions and homogeneity of variance, we performed a log_10_ transformation on parasite spore load.

### (b) Statistical analyses

Statistical analysis was performed using R (R Core Team 2024, v. 4.4.1). First, to assess whether individual infection risk differed given the density of larvae present and parasite dose exposure, we used logistic regression analysis with infection modelled as a binary response (0: uninfected; 1: infected), and density, dose and a density-by-dose interaction as fixed effects. We included plant tube identifier as a random effect to account for non-independence of individuals reared in the same tube. To examine how the total number of infected hosts per experimental unit (tube) was impacted by density and dose, we used a linear regression with number of infected monarchs per tube modelled as a response variable, and density, dose and a density-by-dose interaction as fixed effects. Second, to understand whether spore load is influenced by density and dose, we examined the subset of individuals that became infected and employed a linear mixed-effects model (LMM) with Gaussian errors. Fixed effects included density, parasite dose and a density-by-dose interaction and tube identifier was again a random effect. To examine the impact of dose and density on the total number of spores produced, we used a linear regression with sum of spores for infected hosts per tube modelled as a response variable, and density, dose and a density-by-dose interaction as fixed effects.

**Figure 1.**
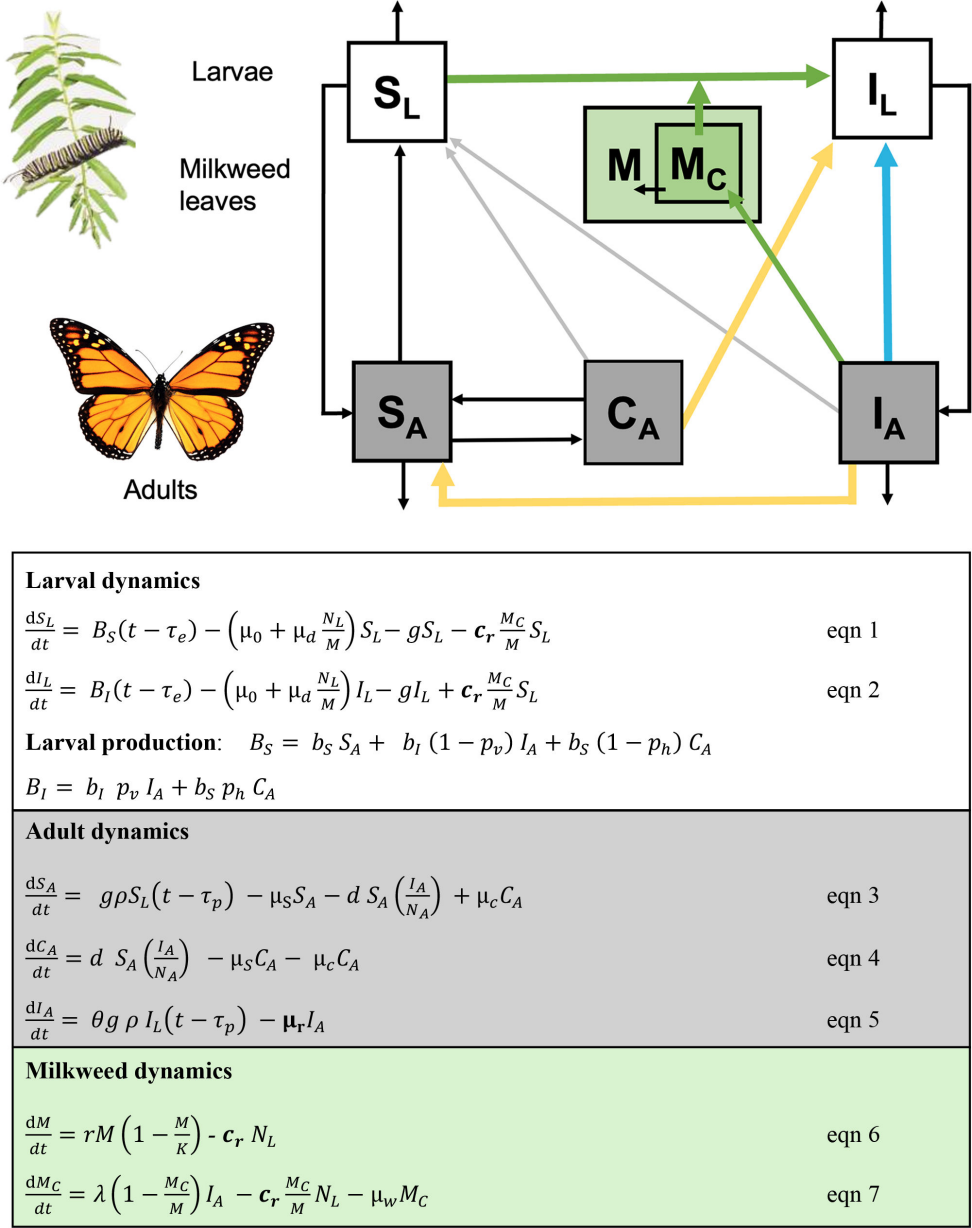
Schematic diagram of the monarch–OE model along with equations. (A) The model is stage and infection structured. *S_L_* and *S_A_* represent abundance of susceptible larvae and adults, respectively. *C_A_* is the abundance of uninfected adults that become contaminated with spores during mating or other close contact. *I_L_* and *I_A_* are infected larval and adult abundances, respectively. Milkweed leaves (*M*), become contaminated (*M_C_*) with OE spores when infected adults deposit spores onto the leaves. Three transmission routes are represented by arrows: vertical transmission (blue line), adult transfer (yellow line) and environmental transmission (green line). (B) White box tracks larval dynamics (*S_L_* = susceptible larvae, *I_L_* = infected larvae), grey box tracks adult dynamics (*S_A_* = susceptible adults; *C_A_* = adults contaminated by spores from other adults, *I_A_* = infected adults) and green box tracks milkweed dynamics (*M* = milkweed; *M_C_* = contaminated milkweed with parasite spores). *N_L_* and *N_A_* are respectively the total number of larvae and adults. Bolded terms with a subscript *r* represent parameters that can be reduced by larval density: consumption rate of milkweed (*c*_*r*_) and mortality rate of infected adults (due to ingestion of lower spore doses)(µ_r_).

**Figure 2.**
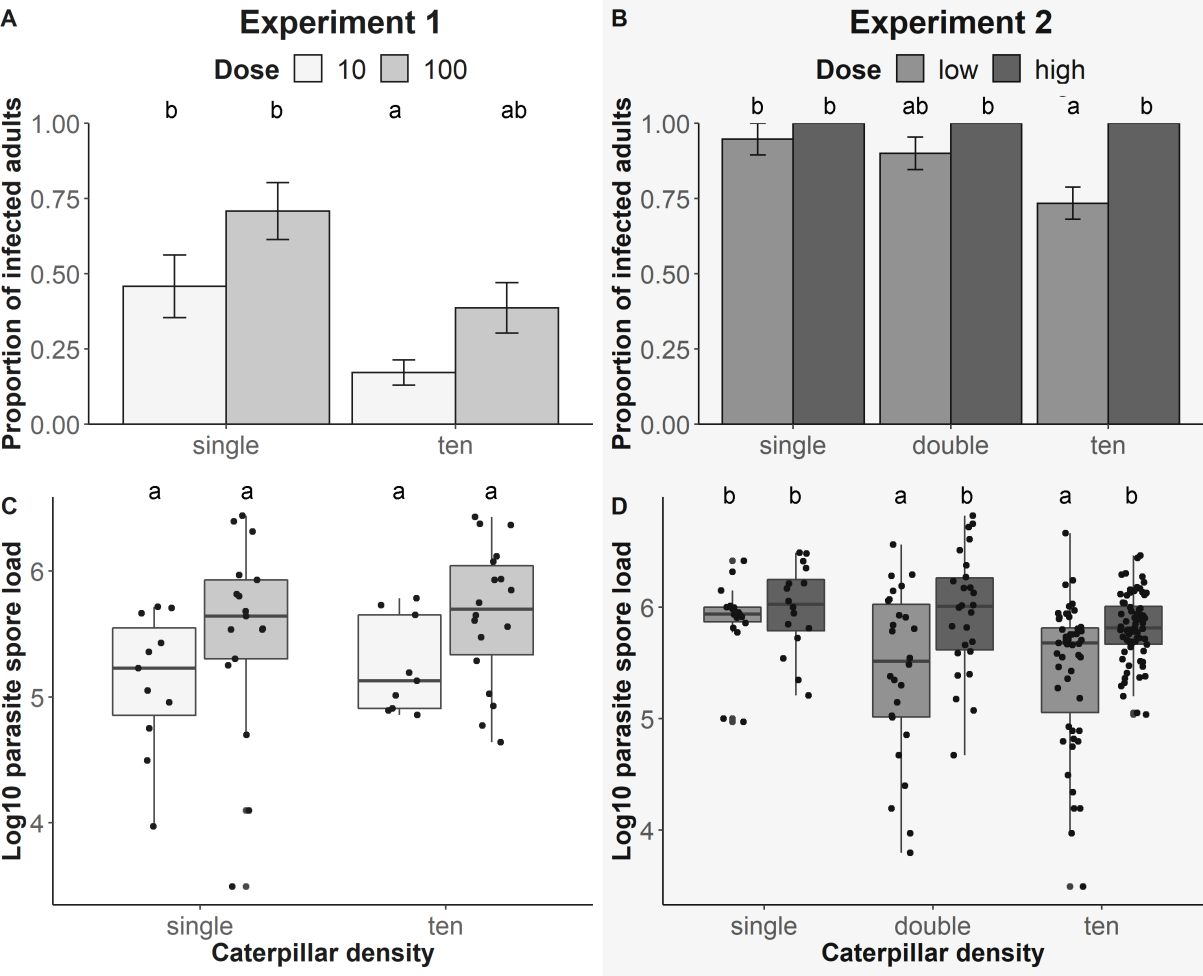
(A,B) Proportion of monarchs that became infected in relation to caterpillar density and parasite dose on rearing plant. (C,D) Log_10_ spore load of infected adults by density and dose. The darker grey fill of bars and boxplots indicates higher exposure dosage. (A,C) Experiment 1 included doses of 10 and 100, while (B,D) experiment 2 included doses of roughly 100 and 1000 spores. Different letters above bars and box plots indicate significant differences among pairwise comparisons (for model outputs see electronic supplementary material, tables S4, S8, S6 and S10).

For all following analyses individuals in the treatment that received a dose of parasites but did not become infected were included as uninfected controls (experiment 1: *n* = 99; experiment 2: *n* = 23). This approach yielded quantitatively similar results to analyses which excluded these individuals altogether (see electronic supplementary material). To assess whether adult lifespan and wing area were impacted by density and dose, we used an LMM with density, parasite dose and a density-by-dose interaction as fixed effects and tube identifier as a random effect. Finally, we performed post hoc pairwise Tukey tests to facilitate interpretation of significant effects presented in figures 1 and 2 (function glht, multcomp package in R [47]).

### (c) Modelling study

We modified an existing stage-structured mechanistic mathematical model of the monarch–OE system [38] to examine the impact of *foraging dilution*. The model tracks the number of susceptible and infected monarch larvae (*S_L_*, *I_L_*), adults (*S_A_*, *I_A_*), the total number of milkweed leaves (*M*) and the number of leaves with an infectious dose of protozoan spores (*M_C_*). The model assumes eggs are produced at per capita rate *b_s_* by susceptible and *b_i_* by infected monarchs. Eggs remain in the egg stage for *t_e_* days before larvae hatch and grow at rate *g*. Larvae experience density-dependent (*µ_0_*) and density-independent mortality rates (*µ_d_*). Larvae become and remain as pupa for *t_g_* days. Pupae have probability (*ρ*) of surviving to adulthood. Susceptible adults experience mortality at rate *µ_S_*. Infected adults have probability of eclosing and mating (*θ*), and experience mortality at rate *µ_I_*.

Three transmission routes are tracked in the model. First, vertical transmission occurs as larvae are infected by spores shed onto their egg cases by their mother. Prior experimental work indicates that infected females infect most (95%) of their larvae [41]. Second, environmental transmission occurs when larvae consume spore-contaminated milkweed leaves *M_C_*. Based on prior field work, we estimated that milkweed grows at rate (*r*) and becomes contaminated as infected adults visit the milkweed and deposit spores at rate (*λ*), or the number of visits that result in deposition of an infectious spore dose [38]. Lastly, adult transfer, or infection of larvae by adults contaminated with spores through mating with infected adults [38], occurs with probability of (*p_h_*). Adults mate at rate *d* and contaminated adults lose spores at rate (*µ_C_*). The model schematic and equations are provided in figure 1, with parameters described in electronic supplementary material, table S11. The model is parameterized for a breeding site with *A. curassavica*, where larval densities tend to be high.

To simulate foraging dilution*,* we made the milkweed (and spore) consumption rate a decreasing function of larval density. The maximum leaf consumption rate is estimated from prior work [48], showing in the absence of other larvae, monarch larvae consume 35 milkweed leaves over the 9 day larval period at 26°C, or 3.89 leaves per day. We assume the consumption rate declines linearly with the total number of larvae *N_L_* per milkweed leaves (*M*), from its maximum value *c* until it reaches zero. Denoting the density-reduced consumption rate as *c_r_*, this can be expressed as


(2.1) $$c_{r}=max\left(c-c_{1}\frac{N_{L}}{M},0\right)$$

where *c_1_* is the strength of the density-dependent reduction in consumption rate (i.e. the reduction in consumption rate from adding 1 larva per leaf).

Prior experimental work shows that lifespan of infected monarchs is negatively impacted by parasite load, where high loads are associated with high adult mortality [36,37,49]. Because reduced leaf consumption is assumed to lower the number of consumed spores, we expect that parasite load and negative impact on infected adult mortality are also reduced. We model this reduction in infected adult mortality by multiplying the maximum infected adult mortality rate by a linearly decreasing function of the consumption reduction (*c_1_N_L_/M*) scaled by a parameter φ; further, we assume that at high host density the mortality rate of infected hosts saturates at the mortality rate of uninfected adults. Denoting the infected mortality rate under consumption reduction, as *µ_r_* this can be written as


(2.2) $$\mu_{r}=max\left(\mu_{I}\left(1-\varphi c_{1}\frac{N_{L}}{M}\right),\mu_{S}\right)$$

We initiated the model with 18 uninfected and 2 infected adult monarchs (to reflect a typical initial abundance of monarch eggs and approx. 10% infection rate in North America [39]) and ran the model for 150 days, based on the length of a breeding season. We explored how the three transmission routes contribute to infection dynamics with density-mediated consumption reduction *c_1_* by calculating the total number of new adult infections arising from each transmission route and the proportion of the total infections attributed to each route as we varied *c_1_*. We used strength of consumption reduction values, *c_1_*, equal to 0, 300, 600, 860 as these correspond to end-of-season leaf consumption rate (*c_r_*) values equal to 3.89, 3, 2 and 1 leaf, respectively (see electronic supplementary material, figure S1). We used R package deSolve [50] to solve the differential equations.

## 3. Results

In both experiments, we observed high survival from caterpillar to adulthood (>88%; electronic supplementary material, tables S3 and S7). In experiment 1, the proportion of infected adults in the single caterpillar treatment with dose 10 was 46% (*n* = 24) and in ten caterpillars with dose 10 was 15% (*n* = 58), while the single caterpillar with dose 100 was 71% (*n* = 24) and in ten caterpillars with dose 100 was 37% (*n* = 49). For exposed caterpillars, infection rates were overall lower in the first experiment across treatments (35%, range: 15–71%) compared with the second experiment (78%, range: 73–100%). In experiment 2, the proportion of infected adults in the single caterpillar treatment with low dose was 95% (*n* = 19), doubles with low dose was 90% (*n* = 29) and ten caterpillars with low dose was 73% (*n* = 70). All caterpillars were infected with high dose, regardless of density in experiment 2 (single: *n* = 16, double: *n* = 26, tens: *n* = 72). No control larvae became infected in this study (electronic supplementary material, tables S3 and S7).

### (a) Experiment 1 (direct spore deposition)

**Figure 3.**
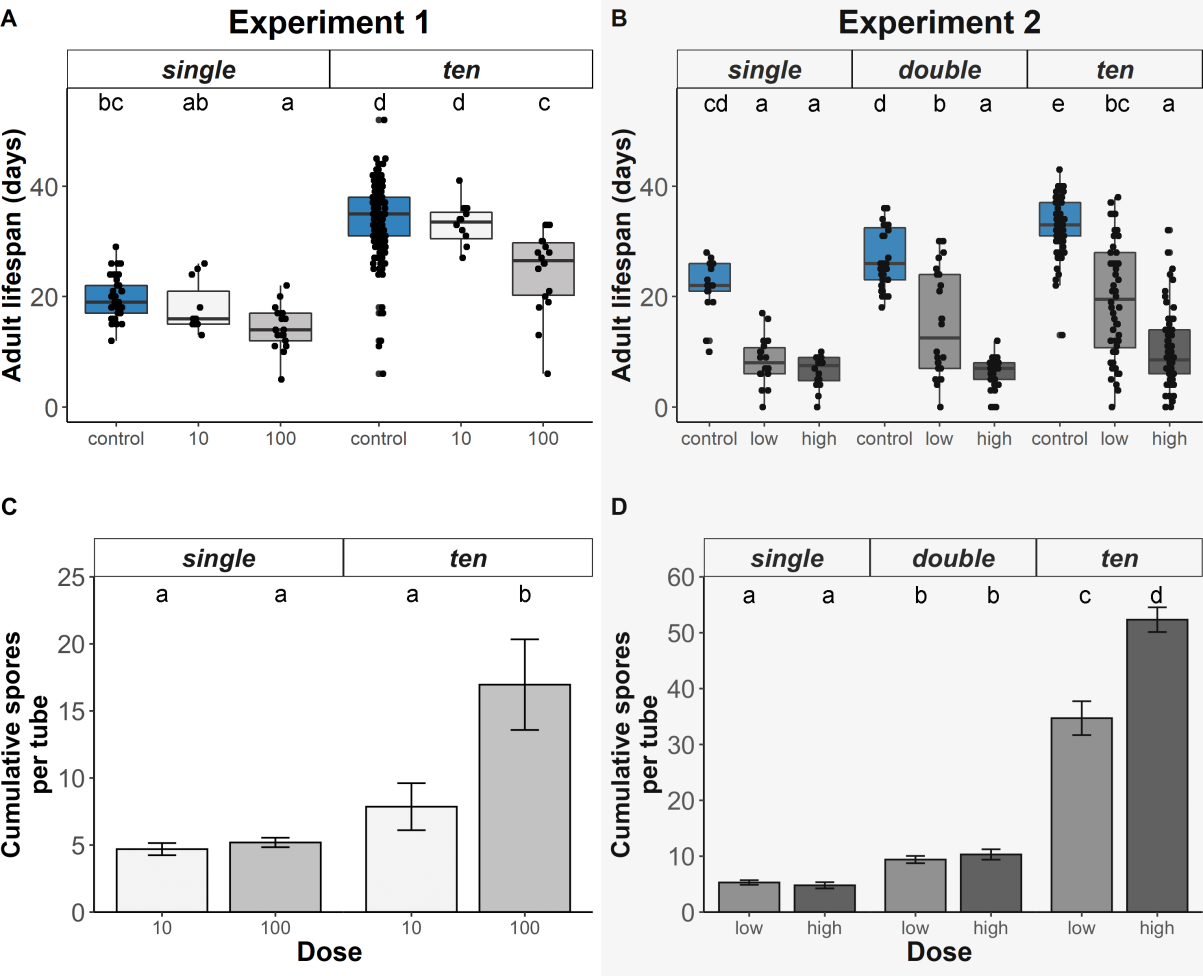
(A,B) Adult lifespan and (C,D) cumulative spore load in relation to caterpillar density (single, double or ten) and parasite dose exposure. Blue represents control monarchs placed on plants with no spores (dose = 0, dose = control) and increasingly darker grey represent the increasing doses of spores caterpillars were exposed to, with (A,C) light greys indicating 10 or 100 spores (experiment 1) and (B,D) dark greys indicating low (approx. 100) or high (approx. 1000) exposure (experiment 2). Different letters above bars and box plots indicate significant differences among pairwise comparisons (for model outputs see electronic supplementary material, tables S5, S6, S9 and S10).

In experiment 1, we controlled the number of spores (dose) deposited on plants and we found that the probability of infection was impacted by caterpillar density and spore dose. When caterpillars shared a host plant with others (i.e. ten caterpillars per plant), their per capita infection risk was lower (*t* = −2.55, df = 36.63, *p* = 0.01; figure 2A). Per capita infection risk was higher on plants inoculated with 100 spores, although this effect was marginally significant (*t* = 1.92, df = 142.80, *p* = 0.06; figure 1A). The total number of infected monarchs per experimental unit (tube) did not differ between dose 10 and 100 (*t* = 0.1, *p* = 0.92) or densities (*t* = 1.63, *p* = 0.11), although we found a significant interaction between dose and density: there were more infected caterpillars in ten caterpillar 100 spore dose exposures (*t* = 3.00, *p* = 0.005). Infected monarchs’ parasite spore loads did not differ between monarchs raised singly or with others, and did not vary with exposure dose (density: *t* = 0.47, df = 29.25, *p* = 0.64; dose: *t* = 1.51, df = 37.21, *p* = 0.14; figure 1C). The total number of spores per experimental unit (tube) did not differ between the 10 and 100 spore doses (*t* = 0.37, *p* = 0.72) or densities (*t* = 1.75, *p* = 0.09) yet we found a significant interaction: the highest total number of spores was found in ten caterpillar 100 dose treatment (*t* = 2.50, *p* = 0.001). Analyses of adult lifespan indicated that monarchs raised on host plants with others lived longer (*t* = 12.72, df = 145.94, *p* < 0.001; figure 3A). Monarchs exposed to 100 spores had shorter adult lifespans compared with other dosages (*t* = −3.31, df = 229.90, *p* < 0.001; figure 3A). Cumulative number of spores on monarchs per tube was not impacted by dose (*t* = 0.37, *p* = 0.72) or density alone (*t* = 1.75, *p* = 0.09). Tubes holding ten caterpillars with 100 dose treatment had higher cumulative spores than other combinations (*t* = 3.45, df = 38, *p* < 0.01; figure 3C). For full model outputs see electronic supplementary material, tables S4 and S5.

### (b) Experiment 2 (spore deposition from infected butterfly)

In the second experiment, spore deposition mimicked more natural exposure to spores deposited by infected females. We found that monarchs raised with conspecifics at high density had lower risk of becoming infected (ten density: *t* = −2.93, df = 225, *p* < 0.01, figure 2B). The exposure dosage of spores on the plant had no overall impact on infection probability in the full statistical model (*p* > 0.05, electronic supplementary material, table S8), but pairwise comparisons showed that caterpillars raised at the high density of ten per plant and exposed to the high dose had lower per-capita infection risk compared with other density–dose combinations (figure 2B, electronic supplementary material, table S10). The total number of infected monarchs per experimental unit (tube) did not differ between low and high doses (*t* = −0.47, *p* = 0.64), but compared with single monarchs, there were more infected in double (*t* = 3.60, *p* < 0.001) and ten caterpillar treatments (*t* = 19.31, *p* < 0.001). There were most infected caterpillars in high dose and ten density treatment compared with all other combinations (*t* = 6.80, *p* < 0.001). Analyses of infected monarchs indicated that caterpillars sharing a plant with others had lower parasite load than caterpillars reared singly (double: *t* = −2.85, df = 159.63, *p* < 0.001; ten: *t* = −2.93, df = 76.21, *p* < 0.001, figure 2D). However, infected monarchs’ spore load was similar when exposed to low and high parasite doses (*t* = 0.06, df = 190.46, *p* = 0.55, figure 2D). Monarchs sharing plants with others lived longer as adults compared with monarchs held singly (double: *t* = 2.56, df = 332.68, *p* = 0.01; ten: *t* = 6.62, df = 227.76, *p* < 0.001, figure 3B). Monarchs exposed to low or high doses lived fewer days as adults than healthy controls (low dose: *t* = −6.39, df = 339.74, *p* < 0.001; high dose: *t* = −7.04, df = 339.74, *p* < 0.001, figure 3B). Cumulative spores per tube was higher among double and tens treatments (double: *t* = 3.05, df = 80, *p* < 0.01; tens: *t* = 17.86, df = 80, *p* < 0.01), but was not influenced by dose (*t* = −0.40, df = 80, *p* = 0.69). Tubes with ten caterpillars and high dose treatment had the highest cumulative number of spores (*t* = 7.78, df = 80, *p* < 0.01; figure 3D). For full model outputs, see electronic supplementary material, tables S9 and S10.

### (c) Modelling

**Figure 4.**
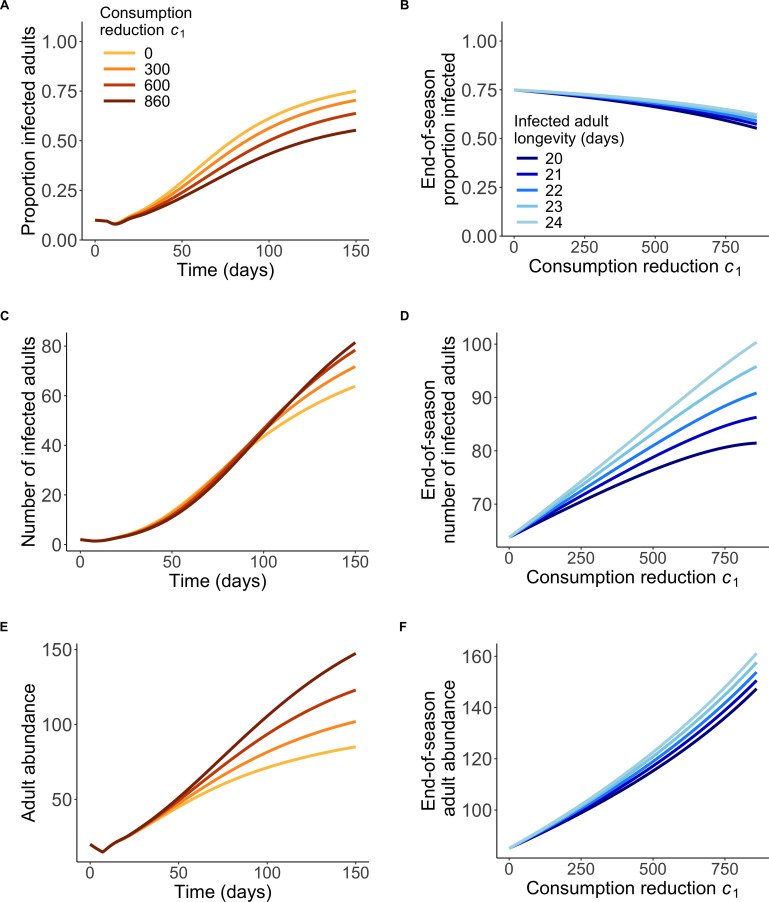
Modelling predictions. (A) Changes in proportion of infected adults, (C) number of infected adults and (E) adult abundance with consumption reduction, *c*_*1*_. Colours of the lines represent different consumption reduction values, with brown colour corresponding to low leaf consumption. End-of-season (B) proportion of infected adults, (D) number of infected adults and (F) adult abundance with increasing infected adult longevity towards that of uninfected adults. Colours of the lines represent days infected monarchs live, with darkest colour representing default longevity.

Numerical solution of the model with no foraging dilution (strength of consumption reduction *c_1_* = 0, leaf consumption rate *c_r_* = 3.89 leaves per monarch per day) showed that the proportion of infected adults increased to a relatively high value (approx. 75%) by the end of the 150 days (figure 4A). As the strength of consumption reduction, *c_1_* increased, the late season per larva leaf consumption rate, *c_r_*, and the cumulative amount of milkweed consumed by all larvae (electronic supplementary material, figure S1 and S2) decreased. Overall, the late season prevalence declined under consumption reduction (figure 4A); however, the total number of infected adults increased (figure 4C), indicating that more transmission occurs under consumption reduction. This reduction in prevalence can be explained by an increase in the overall number of monarchs surviving to adulthood (figure 4E). This is because consumption reduction increases the total amount of milkweed and thus reduces the strength of density-dependent competition on larval mortality (electronic supplementary material, figures S3 and S4). Even though the risk of infection from susceptible larvae encountering and consuming spore-contaminated milkweed goes down under consumption reduction, overall transmission increases because of increased absolute and relative contributions of parent-to-offspring transmission (electronic supplementary material, figure S3e and figure S5). In particular, increases in the number of susceptible monarchs surviving to adulthood under consumption reduction increases the number of susceptible-infected matings, and thus infections of larvae resulting from adult spore transfer. Our results were qualitatively robust to our choice of initial conditions (electronic supplementary material, figure S6); with end-of season prevalence typically declining with an increasing number of colonizing adults and increasing when an increasing infection prevalence of colonizers (electronic supplementary material, figure S7).

Based on our observation of reduced parasite load at high host densities (i.e. experiment 1), we also asked whether the longer-lived infected monarchs would contribute to more new cases and therefore increase infection prevalence under consumption reduction relative to when no consumption reduction occurred. Specifically, we quantified how the reduction in infected adult mortality (*µ_r_*) impacted infection dynamics and adult abundance. Infection prevalence only slightly increased with increased infected adult longevity, but continued to decline with increasing strength of consumption reduction, meaning that reduced virulence relating to lower spore encounter results in shallower declines in prevalence with consumption reduction, but never resulted in prevalence increases (figure 4B). Even though the total number of infected adults strongly increased with increasing infected adult longevity and increasing consumption reduction (figure 4D), the large increases in overall adult abundance under consumption reduction arising from reduced larval competition (figure 4F) countered this effect and maintained the pattern of declining infection prevalence with stronger consumption reduction. This result was robust to variation in the strength of the reduction in virulence associated with spore depletion by conspecifics (electronic supplementary material, figures S8–S10).

Thus, overall, modelling work suggests that the net effect of consumption reduction was to lower infection prevalence but increase overall transmission in spite of reduced per capita infection risk of larvae; this is because reduced larval competition increased adult abundance and parent-to-offspring transmission arising from more susceptible-infected matings.

## 4. Discussion

Our study showed that when many hosts share a food resource that harbours free-living infectious propagules, the individual risk of infection is decreased. Experimental work showed that the reduction in individual-level infection risk is greatest when monarchs are crowded and exposed to a relatively low number of parasite spores (100 or less). Model analysis confirmed that a reduction in food consumption per host under crowding reduced per capita risk of infection risk of susceptible larvae, and lowered population-level infection prevalence. Yet, modelling also showed that the total number of transmission events increased, because greater food availability increased host abundance and increased infections arising from parent-to-offspring transmission routes. Thus, while per-capita infection risk is lowered, the total number of infected hosts increases over the course of the season. Together, these findings suggest that combined effects of host density on resource competition, parasite encounter risk and associated disease induced mortality can have interactive effects on the infection dynamics of pathogens with multiple transmission routes, and lead to opposing outcomes for individual infection risk and population-level transmission.

Experimental work showed that at low parasite exposure crowding reduced individual infection intensity in the host and increased infected host lifespan. However, modelling revealed that increases in transmission related to increased infectious period did not overcome the net effect of consumption reduction in lowering transmission and prevalence. Lowered infection odds due to conspecifics that share a food resource is consistent with the concepts of encounter dilution*,* host foraging interference and friendly competition, where the consumption of resources by competing hosts within a community lowers parasite transmission or parasite load [25], an effect which we call the foraging dilution effect. In the monarch system, crowded caterpillars experience lower per capita infection risk when spores are removed from the plant via leaf consumption by conspecifics, thereby decreasing the likelihood of encountering spores. These results corroborate findings from other host–parasite systems where depletion of infective stages from the environment from co-feeding conspecifics lowers per capita infection risk for other hosts (e.g. crustacean *Daphnia dentifera* and fungal parasite [51]), and intensity (e.g. salmonids and salmon louse [33]).

The results of the experiments suggest that foraging dilution of individual infection risk saturates when the number of infectious propagules in the environment is very high, leading to 100% infection rate. Previous work examining how the density–infection risk relationship in environmentally transmitted pathogen changes over time indicated similar trends [51]. Specifically, when the host *Daphnia dentifera* is at high density, the per capita infection risk with a fungal parasite in the short term is reduced via safety in numbers effect. Yet, in the long term, as additional *Daphnia* generations produce more parasites, the protective effect is lost [51]. Several other systems in which hosts experience safety in numbers show a saturation point of parasites where all hosts experience heightened infection risk [14,29,52]. Overall, our findings add to the growing body of evidence indicating that the density-infection risk relationship is nonlinear. In cases where all monarchs were infected at high host density and high parasite doses (i.e. experiment 2), we observed reductions in parasite intensity per host at the moderate level of exposure (approx. 100 parasite spores). The result suggests that, again, there is a tipping point at which parasite numbers in the environment (i.e. dose) are sufficiently high that all hosts are exposed and infected, and any positive impacts of high density reducing per capita infection risk are nullified. However, crowding still provides a benefit to the host when some parasites are removed from the environment. This mechanism benefits individual hosts by lowering their parasite burden, which has been shown to prolong monarch adult longevity [36], an effect we also observed in this study. Model analysis suggested that decreased mortality of infected adults associated with lower spore intake increased infection prevalence, although the effect was small. Therefore, crowding can simultaneously reduce and increase infection prevalence via different mechanisms, but the net effect in this system was that crowding reduced host infection rate and individual parasite load.

Our results are in agreement with several past studies examining systems with free-living parasite stages, such as trematode parasites and fish or mussels which show a negative association between parasite abundance and host density [31,53]. We suspect that foraging dilution might only operate in some host–parasite systems that experience loss of free-living infectious propagules from the environment. Indeed, systems with complex life cycles, or mobile parasite stages, can show negative relationships between parasite intensity and group size [54].

Interestingly, we found another effect of crowding on monarchs: caterpillars reared with other caterpillars on a plant lived longer as adults compared with singly raised monarchs, as found previously in work examining density impacts on adults [45]. Work in *Drosophila* indicates that crowding at larval stages is a stressor that can extend lifespan by causing hormesis; although the physiological mechanisms responsible for the effect are not fully understood [55,56]. For monarchs, greater adult lifespan results in greater monarch fitness [36], thus one possible explanation is that caterpillars sense the neighbours and ‘prepare’ for resource competition at adulthood (e.g. available milkweed for oviposition, or mates). Yet the strategy is not likely to be favoured because any fitness gains of a longer life are outweighed by the overall risk and cost of OE infection at sites with high densities (e.g. [38]). Indeed, parasitized adult monarchs tend to be poor flyers, and are less likely to mate and lay eggs [57]. In this study, longer lifespan was associated with crowding for controls (uninfected) and lightly infected monarchs only, while very heavily infected monarchs had shortest lifespan, suggesting that at some point parasitism negates the longevity benefits gained with crowding.

Besides foraging dilution, other mechanisms might play a role in the monarch system. In particular, pathogen transmission might be reduced at high densities when less susceptible genotypes clear parasite spores from the environment [25], or differences among individuals in immune function lower the shedding and transmission rate [58]. Additionally, crowding can alter host behaviour, such as avoidance of infected conspecifics [59,60]. In monarchs, crowded conditions in lab experiments can lead to aggressive interactions between late-stage instar caterpillars [61], yet implications of this behaviour for infection dynamics remain unclear.

In conclusion, we demonstrated that high host density can reduce both per capita infection risk and infection intensity via reductions in the effective parasite dose consumed by individual caterpillars, ultimately increasing host fitness. Our results also shed light on the importance of host density for parasite success as the number of transmission events increased with foraging dilution. When doses are relatively low (100 or less, experiment 1), the parasite’s infection success is maximized with higher dose and highest density treatment. Specifically, we observed more infected hosts are produced when dose is low, but density is high, than when dose is high and density is low. Overall, our work suggests that prevalence of an environmentally transmitted parasite can be diminished with crowding when enough free-living propagules are cleared via food consumption. Further, the clearing of parasites likely results in fewer spores being ingested by conspecifics, and ultimately, in lower parasite burdens. However, we also found that foraging dilution is not strong enough to completely reduce individual infection risk, particularly when spore deposition is very high, and the protective effect consumption may provide is overwhelmed. We suspect that when milkweed is limited in the landscape (e.g. urban areas), or when monarchs utilize non-native *Asclepias curassavica* (e.g. sites in southeastern North America), caterpillar densities and spore numbers reach high levels, and despite the clearing of spores, enough remain to result in elevated infection rates [38,44,62,63]. As noted in our experimental results, foraging dilution may only operate when spore loads on plants are relatively low to moderate, which is likely limited to early in the season. Overall, this study highlights the nuances of mechanisms underlying individual infection risk found in wild hosts, particularly when hosts are competitors, and environmental transmission plays a role in the system.

## Data Availability

R code and data are available on Dryad [64]. Supplementary material is available online [65].

## References

[1] Anderson RM, May RM. 1981 The population dynamics of microparasites and their invertebrate hosts. Phil. Trans. R. Soc. B **291**, 451–524. (10.1098/rstb.1981.0005)

[2] Lloyd-Smith JO, Schreiber SJ, Kopp PE, Getz WM. 2005 Superspreading and the effect of individual variation on disease emergence. Nature **438**, 355. (10.1038/nature04153) 16292310 PMC7094981

[3] Langwig KE, Frick WF, Bried JT, Hicks AC, Kunz TH, Marm Kilpatrick A. 2012 Sociality, density‐dependence and microclimates determine the persistence of populations suffering from a novel fungal disease, white‐nose syndrome. Ecol. Lett. **15**, 1050–1057. (10.1111/j.1461-0248.2012.01829.x) 22747672

[4] Farnsworth ML, Wolfe LL, Hobbs NT, Burnham KP, Williams ES, Theobald DM, Conner MM, Miller MW. 2005 Human land use influences chronic wasting disease prevalence in mule deer. Ecol. Appl. **15**, 119–126. (10.1890/04-0194)

[5] Dhondt AA *et al*. 2005 Dynamics of a novel pathogen in an avian host: mycoplasmal conjunctivitis in house finches. Acta Trop. **94**, 77–93. (10.1016/j.actatropica.2005.01.009) 15777638

[6] Knell RJ, Begon M, Thompson DJ. 1996 Transmission dynamics of Bacillus thuringiensis infecting Plodia interpunctella: a test of the mass action assumption with an insect pathogen. Proc. R. Soc. B **263**, 75–81. (10.1098/rspb.1996.0013) 8587898

[7] Dwyer G. 1994 Density dependence and spatial structure in the dynamics of insect pathogens. Am. Nat. **143**, 533–562. (10.1086/285619)

[8] Wolfe MS. 1992 Giardiasis. Clin. Microbiol. Rev. **5**, 93–100. (10.1128/cmr.5.1.93) 1735095 PMC358225

[9] Arneberg P, Skorping A, Grenfell B, Read AF. 1998 Host densities as determinants of abundance in parasite communities. Proc. R. Soc. B **265**, 1283–1289. (10.1098/rspb.1998.0431)

[10] Altizer S *et al*. 2003 Social organization and parasite risk in mammals: integrating theory and empirical studies. Annu. Rev. Ecol. Evol. Syst. **34**, 517–547. (10.1146/annurev.ecolsys.34.030102.151725)

[11] Miller MW, Williams ES, Hobbs NT, Wolfe LL. 2004 Environmental sources of prion transmission in mule deer. Emerg. Infect. Dis. **10**, 1003–1006. (10.3201/eid1006.040010) 15207049 PMC3323154

[12] Storm DJ, Samuel MD, Rolley RE, Shelton P, Keuler NS, Richards BJ, Van Deelen TR. 2013 Deer density and disease prevalence influence transmission of chronic wasting disease in white‐tailed deer. Ecosphere **4**, 1–14. (10.1890/es12-00141.1)

[13] Dallas TA, Krkošek M, Drake JM. 2018 Experimental evidence of a pathogen invasion threshold. R. Soc. Open Sci. **5**, 171975. (10.1098/rsos.171975) 29410876 PMC5792953

[14] Buck JC, Hechinger R, Wood A, Stewart T, Kuris A, Lafferty KD. 2017 Host density increases parasite recruitment but decreases host risk in a snail–trematode system. Ecology **98**, 2029–2038. (10.1002/ecy.1905) 28518406

[15] Johnson PT, Stewart Merrill TE, Dean AD, Fenton A. 2024 Diverging effects of host density and richness across biological scales drive diversity-disease outcomes. Nat. Commun. **15**, 1937. (10.1038/s41467-024-46091-4) 38431719 PMC10908850

[16] Breban R, Drake JM, Stallknecht DE, Rohani P. 2009 The role of environmental transmission in recurrent avian influenza epidemics. PLoS Comput. Biol. **5**, e1000346. (10.1371/journal.pcbi.1000346) 19360126 PMC2660440

[17] Antonovics J. 2017 Transmission dynamics: critical questions and challenges. Phil. Trans. R. Soc. B **372**, 20160087. (10.1098/rstb.2016.0087) 28289255 PMC5352814

[18] Antonovics J, Iwasa Y, Hassell MP. 1995 A generalized model of parasitoid, venereal, and vector-based transmission processes. Am. Nat. **145**, 661–675. (10.1086/285761)

[19] Fenton A, Fairbairn JP, Norman R, Hudson PJ. 2002 Parasite transmission: reconciling theory and reality. J. Anim. Ecol. **71**, 893–905. (10.1046/j.1365-2656.2002.00656.x)

[20] Searle CL *et al*. 2016 Population density, not host competence, drives patterns of disease in an invaded community. Am. Nat. **188**, 554–566. (10.1086/688402) 27788345

[21] Hargrave CW, Hambright KD, Weider LJ. 2011 Variation in resource consumption across a gradient of increasing intra‐ and interspecific richness. Ecology **92**, 1226–1235. (10.1890/09-1948.1) 21797151

[22] Vahl WK, Van der Meer J, Weissing FJ, Van Dullemen D, Piersma T. 2005 The mechanisms of interference competition: two experiments on foraging waders. Behav. Ecol **16**, 845–855. (10.1093/beheco/ari073)

[23] Civitello DJ, Pearsall S, Duffy MA, Hall SR. 2013 Parasite consumption and host interference can inhibit disease spread in dense populations. Ecol. Lett. **16**, 626–634. (10.1111/ele.12089) 23452184

[24] Mooring MS, Hart BL. 1992 Animal grouping for protection from parasites: selfish herd and encounter-dilution effects. Behaviour **123**, 173–193. (10.1163/156853992x00011)

[25] Hall SR, Becker CR, Simonis JL, Duffy MA, Tessier AJ, Cáceres CE. 2009 Friendly competition: evidence for a dilution effect among competitors in a planktonic host–parasite system. Ecology **90**, 791–801. (10.1890/08-0838.1) 19341148

[26] Cáceres C, Davis G, Duple S, Hall S, Koss A, Lee P, Rapti Z. 2014 Complex Daphnia interactions with parasites and competitors. Math. Biosci. **258**, 148–161. (10.1016/j.mbs.2014.10.002) 25445737

[27] Dallas T, Hall RJ, Drake JM. 2016 Competition‐mediated feedbacks in experimental multispecies epizootics. Ecology **97**, 661–670. (10.1890/15-0305.1) 27197393

[28] Valois AE, Burns CW. 2016 Parasites as prey: Daphnia reduce transmission success of an oomycete brood parasite in the calanoid copepod Boeckella. J. Plankton Res. **38**, 1281–1288. (10.1093/plankt/fbw055)

[29] Rifkin JL, Nunn CL, Garamszegi LZ. 2012 Do animals living in larger groups experience greater parasitism? A meta-analysis. Am. Nat. **180**, 70–82. (10.1086/666081) 22673652

[30] Cote IM, Poulin R. 1995 Parasitism and group size in social animals: a meta-analysis. Behav. Ecol. **6**, 159–165. (10.1093/beheco/6.2.159)

[31] Buck JC, Lutterschmidt WI. 2017 Parasite abundance decreases with host density: evidence of the encounter-dilution effect for a parasite with a complex life cycle. Hydrobiologia **784**, 201–210. (10.1007/s10750-016-2874-8)

[32] Clay PA, Cortez MH, Duffy MA. 2021 Dose relationships can exacerbate, mute, or reverse the impact of heterospecific host density on infection prevalence. Ecology **102**, e03422. (10.1002/ecy.3422) 34086356

[33] Samsing F, Oppedal F, Johansson D, Bui S, Dempster T. 2014 High host densities dilute sea lice Lepeophtheirus salmonis loads on individual Atlantic salmon, but do not reduce lice infection success. Aquac. Environ. Interact. **6**, 81–89. (10.3354/aei00118)

[34] Fauchald P, Rødven R, Bårdsen BJ, Langeland K, Tveraa T, Yoccoz NG, Ims RA. 2007 Escaping parasitism in the selfish herd: age, size and density‐dependent warble fly infestation in reindeer. Oikos **116**, 491–499. (10.1111/j.0030-1299.2007.15390.x)

[35] Bradley CA, Altizer S. 2005 Parasites hinder monarch butterfly flight: implications for disease spread in migratory hosts. Ecol. Lett. **8**, 290–300. (10.1111/j.1461-0248.2005.00722.x)

[36] de Roode JC, Chi J, Rarick RM, Altizer S. 2009 Strength in numbers: high parasite burdens increase transmission of a protozoan parasite of monarch butterflies (Danaus plexippus). Oecologia **161**, 67–75. (10.1007/s00442-009-1361-6) 19418070

[37] de Roode JC, Gold LR, Altizer S. 2007 Virulence determinants in a natural butterfly–parasite system. Parasitology **134**, 657–668. (10.1017/S0031182006002009) 17140464

[38] Majewska AA, Sims S, Schneider A, Altizer S, Hall RJ. 2019 Multiple transmission routes sustain high prevalence of a virulent parasite in a butterfly host. Proc. R. Soc. B **286**, 20191630. (10.1098/rspb.2019.1630) PMC6742984 31480975

[39] Majewska AA, Davis AK, Altizer S, de Roode JC. 2022 Parasite dynamics in North American monarchs predicted by host density and seasonal migratory culling. J. Anim. Ecol. **91**, 780–793. (10.1111/1365-2656.13678) 35174493

[40] McLaughlin R, Myers J. 1970 Ophryocystis elektroscirrha sp. n., a neogregarine pathogen of the monarch butterfly Danaus plexippus (L.) and the Florida queen butterfly D. gilippus berenice Cramer. J. Eukaryot. Microbiol. **17**, 300–305. (10.1111/j.1550-7408.1970.tb02375.x)

[41] Altizer S, Oberhauser KS, Geurts KA. 2004 Transmission of the protozoan parasite, *Ophryocystis elektroscirrha*, in monarch butterfly populations: implications for prevalence and population-level impacts. In The monarch butterfly: biology and conservation (eds KS Oberhauser, M Solensky), pp. 203–218. Ithaca, NY: Cornell University Press.

[42] Leong KL, Kaya HK, Yoshimura MA, Frey DF. 1992 The occurrence and effect of a protozoan parasite, Ophryocystis elektroscirrha (Neogregarinida: Ophryocystidae) on overwintering monarch butterflies, Danaus plexippus (Lepidoptera: Danaidae) from two California winter sites. Ecol. Entomol. **17**, 338–342. (10.1111/j.1365-2311.1992.tb01067.x)

[43] Nail KR, Stenoien C, Oberhauser KS. 2015 Immature monarch survival: effects of site characteristics, density, and time. Ann. Entomol. Soc. Am. **108**, 680–690. (10.1093/aesa/sav047)

[44] Satterfield DA, Maerz JC, Altizer S. 2015 Loss of migratory behaviour increases infection risk for a butterfly host. Proc. R. Soc. B **282**, 20141734. (10.1098/rspb.2014.1734) PMC4308991 25589600

[45] Alaidrous W, Villa SM, de Roode JC, Majewska AA. 2022 Crowding does not affect monarch butterflies’ resistance to a protozoan parasite. Ecol. Evol. **12**, e8791. (10.1002/ece3.8791) 35414899 PMC8986514

[46] De Anda A, Oberhauser K, Nail K, Altizer S. 2015 Invertebrate natural enemies and stage-specific mortality rates of monarch eggs and larvae. In Monarchs in a changing world: biology and conservation of an iconic butterfly (eds K Oberhauser, K Nail, S Altizer), pp. 60–70. Ithaca, NY: Cornell University Press. (10.7591/9780801455605)

[47] Hothorn T, Bretz F, Westfall P, Heiberger RM, Schuetzenmeister A, Scheibe S. 2017 multcomp: simultaneous inference in general parametric models. v 1.4-19. See https://multcomp.r-forge.r-project.org/.

[48] Satterfield DA, Altizer S, Williams MK, Hall RJ. 2017 Environmental persistence influences infection dynamics for a butterfly pathogen. PLoS One **12**, e0169982. (10.1371/journal.pone.0169982) 28099501 PMC5242512

[49] Altizer S. 2001 Migratory behaviour and host–parasite co-evolution in natural populations of monarch butterflies infected with a protozoan parasite. Evol. Ecol. Res. **3**, 567–581.

[50] Soetaert K, Petzoldt T, Setzer RW. 2010 Solving differential equations in R: package deSolve. J. Stat. Softw **33**, 1–25. (10.18637/jss.v033.i09)20808728

[51] Stewart Merrill TE, Cáceres CE, Gray S, Laird VR, Schnitzler ZT, Buck JC. 2022 Timescale reverses the relationship between host density and infection risk. Proc. R. Soc. B **289**, 20221106. (10.1098/rspb.2022.1106) PMC9346366 35919996

[52] Song Z, Proctor H. 2020 Parasite prevalence in intermediate hosts increases with waterbody age and abundance of final hosts. Oecologia **192**, 311–321. (10.1007/s00442-020-04600-4) 32006182

[53] Levakin IA, Nikolaev KE, Galaktionov KV. 2023 The effect of host density on parasite infection: a power-law model and an experimental test with trematode larvae infecting mussels. Mar. Biol. **170**, 13. (10.1007/s00227-022-04162-4)

[54] Patterson JE, Ruckstuhl KE. 2013 Parasite infection and host group size: a meta-analytical review. Parasitology **140**, 803–813. (10.1017/s0031182012002259) 23425516 PMC3638372

[55] Gems D, Partridge L. 2008 Stress-response hormesis and aging: ‘that which does not kill us makes us stronger’. Cell Metab. **7**, 200–203. (10.1016/j.cmet.2008.01.001) 18316025

[56] Klepsatel P, Procházka E, Gáliková M. 2018 Crowding of Drosophila larvae affects lifespan and other life-history traits via reduced availability of dietary yeast. Exp. Gerontol. **110**, 298–308. (10.1016/j.exger.2018.06.016) 29932967

[57] Altizer SM, Oberhauser KS. 1999 Effects of the protozoan parasite Ophryocystis elektroscirrha on the fitness of monarch butterflies (Danaus plexippus). J. Invertebr. Pathol. **74**, 76–88. (10.1006/jipa.1999.4853) 10388550

[58] Keesing F, Ostfeld RS. 2021 Dilution effects in disease ecology. Ecol. Lett. **24**, 2490–2505. (10.1111/ele.13875) 34482609 PMC9291114

[59] Johnson MS, Johnson LL. 1991 Female choice of males with low parasite loads in sage grouse. In Bird–parasite interactions: ecology, evolution, and behaviour (eds J Loye, M Zuk), pp. 377–388. Oxford, UK: Oxford University Press. (10.1093/oso/9780198577386.003.0020)

[60] Kiesecker JM, Skelly DK, Beard KH, Preisser E. 1999 Behavioral reduction of infection risk. Proc. Natl Acad. Sci. USA **96**, 9165–9168. (10.1073/pnas.96.16.9165) 10430913 PMC17750

[61] Collie J, Granela O, Brown EB, Keene AC. 2020 Aggression is induced by resource limitation in the monarch caterpillar. iScience **23**, 101791. (10.1016/j.isci.2020.101791) 33376972 PMC7756136

[62] Satterfield DA, Villablanca FX, Maerz JC, Altizer S. 2016 Migratory monarchs wintering in California experience low infection risk compared to monarchs breeding year-round on non-native milkweed. Integr. Comp. Biol. **56**, 343–352. (10.1093/icb/icw030) 27252207

[63] Majewska AA, Satterfield DA, Harrison RB, Altizer S, Hepinstall-Cymerman J. 2019 Urbanization predicts infection risk by a protozoan parasite in non-migratory populations of monarch butterflies from the southern coastal US and Hawaii. Landsc. Ecol. **34**, 649–661. (10.1007/s10980-019-00799-7)

[64] Majewska AA, Hall R, De Roode J. 2025 Data from: Crowding reduces per capita parasite infection risk in a butterfly host. Dryad Digital Repository. (10.5061/dryad.8sf7m0czp)40925566

[65] Majewska AA, Hall RJ, De Roode J. 2025 Supplementary material from: Crowding reduces per-capita parasite infection risk in a butterfly host. Figshare. (10.6084/m9.figshare.c.8003643)40925566

